# Galectin‐1, Adiponectin, Leptin, and the Adiponectin to Leptin Ratio as Predictors of Metabolic Syndrome: Influence of Age and Body Mass Index

**DOI:** 10.1155/bmri/1789457

**Published:** 2026-04-06

**Authors:** Darya Saeed Abdulateef, Trifa Abdalla Mahmood, Lizan Ismail Arkawazi

**Affiliations:** ^1^ Department of Basic Medical Sciences, College of Medicine, University of Sulaimani, Sulaymaniyah, Iraq, univsul.edu.iq

**Keywords:** adiponectin, age group, body mass index, galectin-1, leptin, metabolic syndrome

## Abstract

**Background:**

Metabolic syndrome (MetS) is a multifactorial condition linked to adipose tissue dysfunction and insulin resistance. Emerging evidence highlights the potential roles of adipokines leptin, adiponectin, and galectin‐1 in its pathogenesis. However, studies examining these biomarkers as predictors of MetS, particularly in the context of age and body mass index (BMI), remain limited and inconsistent.

**Objective:**

The objective of this study is to evaluate the association of serum galectin‐1, leptin, adiponectin, and the adiponectin/leptin ratio (A/L) with MetS, and to identify their predictive value across different age and BMI groups.

**Methods:**

In this prospective cross‐sectional study, 122 participants were enrolled and categorized into MetS (*n* = 48) and healthy control (*n* = 74) groups based on NHLBI/AHA criteria. Anthropometric, clinical, and biochemical parameters were recorded. Serum levels of galectin‐1, leptin, and adiponectin were measured, and the A/L was calculated. Participants were stratified by age (20–39 vs. ≥ 40 years) and BMI (normal, overweight, and obese). Group comparisons, correlation analysis, and binary logistic regression were conducted.

**Results:**

Galectin‐1 and adiponectin levels were significantly lower in the MetS group, whereas leptin levels were not significantly higher. The A/L ratio was significantly lower in the MetS group, particularly among middle‐aged and obese individuals (*p* = 0.004). Galectin‐1 correlated positively with adiponectin and the A/L ratio. In logistic regression analysis, A/L emerged as the only independent predictor, with each unit increase reducing the odds of MetS by 8.3%, whereas BMI was a consistent predictor across age groups.

**Conclusion:**

Galectin‐1, adiponectin, and the A/L ratio are associated with MetS, with the A/L serving as a robust independent predictor. These findings underscore the importance of adipokine profiling, particularly in stratified age and BMI groups, for early identification of metabolic risk.

## 1. Introduction

Metabolic syndrome (MetS) is a complex disorder influenced by multiple factors originating from insulin resistance in conjunction with abnormal fat distribution and adipose tissue dysfunction [1, 2]. The etiology of MetS has not been fully identified [3]. However, multiple risk factors have been identified, including a family history of the condition, unhealthy dietary habits, such as high‐calorie, low‐fiber fast food, and insufficient physical activity resulting from sedentary lifestyles, all of which contribute to the global burden of MetS [4].

Factors that contribute to MetS include insulin resistance and impaired adipose tissue function [5] that is a key contributor to the development of insulin resistance associated with obesity. Enlargement of adipocytes and macrophage infiltration into adipose tissue lead to the secretion of proinflammatory cytokines, which in turn, exacerbate insulin resistance [6]. The rising prevalence of MetS is closely associated with the increasing rates of obesity [3, 7].

Nowadays, adipose secreted biomarkers, serum levels of galectin, adiponectin, and leptin are found to have correlations with MetS and its indices. Fecal galectin‐1 is associated with MetS in patients with ulcerative colitis, with a protective and immunosuppressive effect [8].

Galectin‐1 is also associated with metabolic indices in individuals with hepatic manifestation of MetS [9]. Galectin‐3 has an association with obesity and MetS in Chinese adolescents [10]. Both galectin‐1 and ‐3 are found to have a correlation with MetS parameters, and both have overlapping effects [11]. Leptin is also found to play an important role in the pathophysiology of MetS as high leptin is associated with obesity; in contrast, another study in T2DM reported no change in leptin or adiponectin [12].

Studies found lower adiponectin and higher leptin levels in individuals with MetS, as well as low levels of adiponectin associated with a decrease in insulin sensitivity, an increase in BMI, an increase in inflammatory markers, and a less favorable lipid profile [13]. Although no association of adiponectin with MetS was found in the Iranian population [14]. More recently, another study found that the L/A ratio was associated with blood pressure and other metabolic profiles, providing an important complement to HOMA‐IR for the risk of MetS, and suggested as a good marker to assess MetS [15].

Previous studies have reported inconsistent findings regarding the association of adiponectin, leptin, and their ratio with MetS [12–14] . In contrast, evidence on the association between serum galectin‐1 and MetS is limited [11], with most studies focusing on its relationship with individual components of the syndrome [8]. other galectins such as galectin‐3 [10], fecal galectin‐1 [9], or populations with specific comorbid conditions, including ulcerative colitis [9]. Furthermore, research on adipokines, particularly leptin, adiponectin, and their ratio, has often examined associations with isolated metabolic parameters and frequently without adequate adjustment for important confounders such as age and BMI [12–14].

Therefore, the aim of this study is to comprehensively evaluate the association of serum galectin‐1, leptin, adiponectin, and the adiponectin/leptin ratio with MetS in a general population, and to identify independent predictors of MetS after adjusting for age and BMI.

## 2. Patients and Methods

### 2.1. Study Design and Setting

A prospective cross‐sectional study was carried out among 122 consecutive individuals (74 healthy individuals and 48 MetS patients), including patients′ accomplices, staff, or volunteers, from July to September 2025 at Smart Health Tower, Sulaimaniyah, Iraq.

### 2.2. Group Definition

Participants were divided into MetS or non‐MetS (control) groups according to guidelines from the National Heart, Lung, and Blood Institute (NHLBI) and the American Heart Association (AHA). MetS was diagnosed when a patient has at least three of the following conditions [3]:

• Fasting blood glucose (FBG) of ≥ 100 mg/dL (or receiving drug therapy for hyperglycemia)
• Blood pressure of ≥ 130/85 mmHg (or receiving drug therapy for hypertension)
• Triglycerides (TG) of ≥150 mg/dL (or receiving drug therapy for hypertriglyceridemia)
• HDL‐C of < 40 mg/dL in men or < 50 mg/dL in women (or receiving drug therapy for reduced HDL‐C)
• Waist circumference of ≥ 102 cm (40 in) in men or ≥ 88 cm (35 in) in women.

Furthermore, patients were allocated to the young age group (20–39 years) and middle age group (> 40 years), whereas BMI groups were considered as normal weight (18–24.9 kg/m^2^), overweight (25–29.9 kg/m^2^), and obese (≥ 30 kg/m^2^).

#### 2.2.1. Inclusion Criteria

Individuals with a confirmed diagnosis of MetS, but no other comorbid conditions.

#### 2.2.2. Exclusion Criteria

Individuals with acute or chronic medical conditions that could influence the study results, especially those who had a history of T1DM and T2DM, liver or kidney diseases, active cancer, or who had undergone significant weight loss interventions. Additional exclusion factors included adherence to a specific diet, substantial body weight changes within the past year, or any chronic illness that might interfere with study outcomes.

### 2.3. Study Protocol

A detailed questionnaire with medical, surgical history was filled for each participant. The eligible participants were further analyzed for anthropometric measures (height, weight, and waist circumference) and blood pressure measurement. Weight was assessed in the morning under fasting conditions, with participants wearing light clothing and no footwear. BMI and waist to height ratio (WHtR) were then calculated accordingly. Blood pressure measurements were conducted using an automated electronic sphygmomanometer while participants were in a seated position after at least 10 min of rest. Both systolic blood pressure (SBP) and diastolic blood pressure (DBP) values were recorded in millimeters of mercury (mmHg). To ensure accuracy, measurements were at heart level, and if necessary, repeated with the average of two readings used for analysis.

### 2.4. Laboratory Investigations

Morning venous blood samples (6.0 mL) were obtained from participants following an overnight fast. Glycated hemoglobin (HbA1c), FBG, and fasting plasma insulin (FPI) levels were measured. Fasting serum levels of glucose, TG, total cholesterol (TC), low‐density lipoprotein (LDL), and high‐density lipoprotein (HDL) were also measured using an enzymatic method on the COBAS Proc503 module system (Roche Diagnostics, GmbH, Germany). The separated serum samples were stored at −80°C for later measurement of galectin‐1, adiponectin, and leptin levels. Quantification of galectin‐1 (Catalog No. E298Hu), adiponectin (Catalog No. E1550llu), and leptin (Catalog No. E1559llu) was performed using enzyme‐linked immunosorbent assay (ELISA) kits obtained from Bioassay Technology Laboratory (Shanghai, China). The ELISA assays demonstrated sensitivities of 0.15 ng/mL for galectin‐1, 0.11 mg/L for adiponectin, and 0.021 ng/mL for leptin. The standard curve ranges were 0.3–90 ng/mL for galectin‐1, 0.2–60 mg/L for adiponectin, and 0.05–10 ng/mL for leptin. The intra‐assay and interassay coefficients of variation were < 8% and 10%, respectively. The HOMA‐IR, an index of hepatic insulin resistance, was calculated using the known equation, which is generated by fasting plasma glucose (FPG) × FPI. HOMA‐IR = I0 ∗ G0/22.5, normally < 2.5 [16]. HOMA − B (*%*) = (20 × fasting insulin in *μ*U/mL)/(fasting glucose in mmol/L − 3.5) [17].

### 2.5. Ethical Approval

The research protocol received approval from the Ethics Committee of the College of Medicine, University of Sulaimani, Sulaymaniyah, Iraq (No.148/13 on July 13, 2025). The written informed consent was obtained from each participant.

### 2.6. Statistical Analysis

The collected Excel sheet data were imported into SPSS, Version 26 (IBM, Chicago, United States). Descriptive statistics were performed, and the parametric and nonparametric data were defined using normality tests. Parametric variables were compared using the Student′s *T*‐test and presented as mean ± standard deviation (SD), whereas nonparametric variables were analyzed using the Mann–Whitney test and expressed as median. Spearman′s linear correlations were used to find the association between variables. Binary logistic regression analysis was performed to identify parameters associated with increased odds of having MetS. A *p* ≤ 0.05 was considered statistically significant, whereas *p* < 0.001 was set as highly significant.

## 3. Results

All lipid profiles, glycemic indices, blood pressure parameters, and waist circumference are significantly higher in the MetS group compared with healthy controls, whereas serum HDL and insulin show higher levels among healthy controls (*p* ≤ 0.05). No significant difference exists in the average pulse rate between the two studied groups. (Table 1).

**Table 1:** Comparative analysis of parameters in metabolic syndrome and control groups.

Variable	Control *m* *e* *a* *n* ± *S* *D* median [range] (*n* = 74)	Metabolic syndrome *m* *e* *a* *n* ± *S* *D* median [range] (*n* = 48)	*p*
Galectin (mg/L)	0.292 [0.007–11.421]	0.205 [0.005–4.415]	0.038∗
Adiponectin (mg/L)	3.291 [0.015–27.782]	1.849 [0.176–17.296]	0.015∗
Leptin (ng/mL)	0.322 [0.000–3.979]	0.328 [0.086–1.602]	0.543
Adiponectin/leptin ratio	10.75 [0.39–65.10]	7.97 [0.42–128.12]	0.079
Total cholesterol (mg/dL)	175.98 (34.41)	191.81 (45.41)	0.031∗
Triglyceride (mg/dL)	100.2 [48.0–310.6]	190.1 [76.6–501.0]	< 0.001∗∗
High‐density lipoprotein (mg/dL)	50.0 [33.6–95.0]	38.35 [27.0–81.0]	< 0.001∗∗
Low‐density lipoprotein (mg/dL)	112.27 (29.07)	124.92 (40.32)	0.047∗
Cholesterol/HDL ratio	3.64 (0.95)	4.87 (1.22)	< 0.001∗∗
Fasting plasma glucose (mg/dL)	98.5 [85.6–132.0]	104.1 [86.1–238.0]	< 0.001∗∗
HbA1c (%)	5.51 [4.56–6.4]	5.77 [4.81–7.85]	< 0.001∗∗
Insulin (mU/L)	10.71 [2.13–50.20]	10.49 [4.84–52.90]	< 0.001∗∗
HOMA‐IR	2.62 [0.45–16.35]	4.39 [1.09–16.58]	< 0.001∗∗
HOMA‐B (%)	113.69 [32.52–291.92]	135.71 [24.07–488.31]	0.035∗
Pulse rate (bpm)	78.57 (10.28)	80.13 (10.08)	0.412

*Note:* Single asterisk denotes significant difference; double asterisks denote highly significant difference. Parametric variables were compared using the Student′s *T*‐test and presented as mean ± SD, whereas nonparametric variables were analyzed using the Mann–Whitney test and expressed as median.

**Figure 1:**
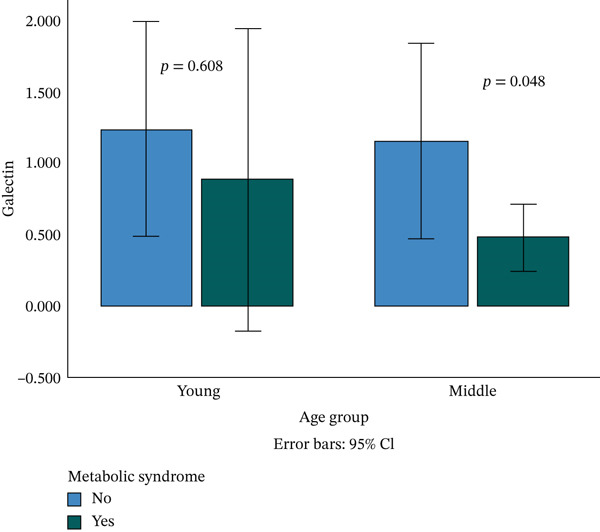
Comparison of serum galectin between metabolic syndrome and control in both young and middle age groups.

**Figure 2:**
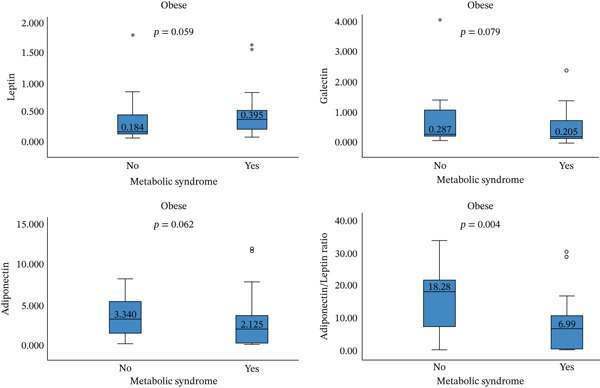
Comparison of galectin‐1, leptin, adiponectin, and adiponectin/leptin ratio between metabolic syndrome and control obese participants.

Serum galectin‐1 and adiponectin are significantly lower among MetS subjects compared with healthy subjects, with *p* = 0.038 and *p* = 0.015, respectively. Higher serum leptin and lower serum adiponectin/leptin (A/L) are found among MetS individuals, but the results failed to reach the significant level. When the participants are stratified by age group, the significant value remains for the middle age group (*p* = 0.048) (Figure 1). Stratification by the BMI group is demonstrated in Figure 2, galectin‐1 and adiponectin are lower and leptin is higher in the MetS group, but they did not reach the significant level, whereas the A/L ratio is significantly lower in MetS compared with the control group (*p* = 0.004).

A significant correlation between serum galectin‐1, adiponectin, leptin, and A/L with a significant negative correlation between serum adiponectin and TG, A/L, and HbA1c, and a significant positive correlation of A/L and HOMA‐B were seen (Figure 3).

Figure 3Correlations between adiponectin and triglycerides (a) and adiponectin/leptin ratio and HbA1c (b) and HOMA‐B (c).

**Figure:**
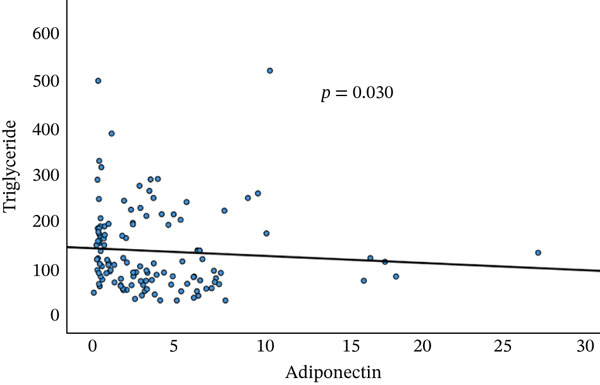
(a)

**Figure:**
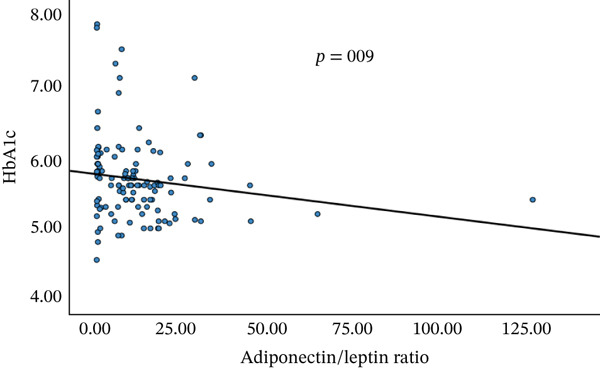
(b)

**Figure:**
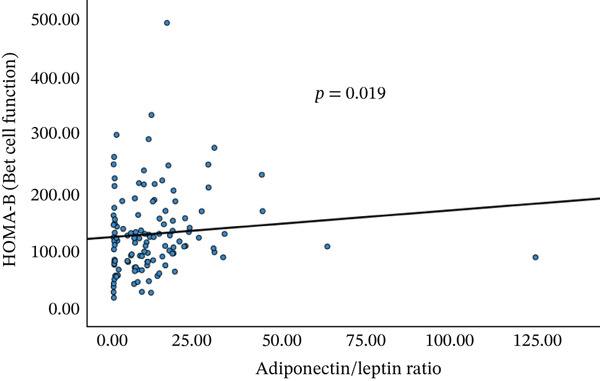
(c)

Significant correlation of MetS indices, subjective perceived stress, serum galectin‐1, and adipokines among healthy and MetS groups are demonstrated in Figure 4. In the MetS group, among the measured serum galectin‐1 and adipokines, serum leptin showed a significant positive correlation with HbA1c. No other statistically significant correlation between serum galectin‐1, adipokines, and MetS indices is found. In the control group, no statistically significant correlations were identified.

Table 2 illustrates the significant correlations of parameters with MetS among different age and BMI groups. In the young age group, only BMI has significant correlation with the presence of MetS, whereas in the middle age group, both BMI and galectin‐1 have significant association with the presence of MetS. In the young obese and middle age obese groups, the only parameters that have significant correlation with the presence of MetS are A/L.

**Table 2:** Significant correlation of parameters with the presence of metabolic syndrome.

Groups	Young age	Middle age		Young obese	Middle age obese
Metabolic syndrome	BMI	BMI	Galectin	Adiponectin/leptin ratio	Adiponectin/leptin ratio
*ρ*	0.408	0.318	0.221	−0.619	−0.351
*p* value	0.008∗	0.004∗	0.048∗	0.024∗	0.042∗

*Note:* Asterisk denotes significant difference.

Abbreviation: BMI, body mass index.

When parameters are analyzed via binary logistic regression analysis to find the odds of having MetS among both young and middle age group, only BMI increases the odds of having MetS. A/L is the only significant predictor; with every one‐unit increase in this ratio, the odds of having MetS decrease by 8.3% (Table 3).

**Table 3:** Binary logistic regression analysis, with the presence of metabolic syndrome as outcome variable in different patient groups.

Model	Unstandardized coefficients		Odds ratio exp. (B)	*p*	95% CI for B	
					Lower	Upper
	B	SE			Bound	
Young age (*n* = 41)						
Galectin	−0.201	0.204	0.818	0.326	0.548	1.221
BMI	0.304	0.125	1.355	0.016∗	1.059	1.733
Middle age (*n* = 81)						
Galectin	0.0388	0.248	0.678	0.117	0.417	1.103
BMI	0.145	0.057	1.156	0.010∗	1.035	1.292
Young obese (*n* = 13)						
Adiponectin/leptin ratio	−0.213	0.126	0.808	0.091	0.631	1.035
Middle age obese (*n* = 34)						
Adiponectin/leptin ratio	−0.087	0.044	0.917	0.046∗	0.842	0.999

*Note:* Presence of metabolic syndrome codding as 0 = no metabolic syndrome and 1 = metabolic syndrome. Asterisk denotes significant difference.

## 4. Discussion

This study examined the relationship between galectin‐1, adipokines, and the A/L with MetS. We found lower levels of galectin‐1 and adiponectin in individuals with MetS, whereas leptin was higher but not significant. The A/L was the strongest and most consistent predictor, independent of age and BMI. These findings highlight the importance of adipokine balance as a marker of metabolic risk and provide a basis for comparison with previous studies.

In the present study, serum galectin‐1 was significantly lower in MetS, consistent with findings of Fryk et al. that found this outcome for T2DM [18], but contrasting with studies in Chinese and Swedish populations that reported higher levels [9, 11]. These discrepancies may reflect differences in population characteristics (age, obesity, and comorbidities) or disease stage. Furthermore, Miwa et al. [19] observed a negative association between MetS parameters and serum adiponectin levels. Additionally, a meta‐analysis reported that the presence of MetS is linked to reduced adiponectin levels [20]. However, Esfanjani et al. found no significant relationship between adiponectin and MetS in an Iranian population [14].

Although the present study observed higher serum leptin levels and a lower A/L ratio among individuals with MetS, these differences were not statistically significant. Similar findings have been reported, showing that leptin levels did not differ significantly between MetS cases and healthy individuals [21]. Whereas previous studies have consistently highlighted the role of leptin and adiponectin in obesity and metabolic disease development. A 2019 review reported that elevated leptin concentrations are directly associated with obesity and its subsequent metabolic complications [12]. Similarly, López et al. demonstrated that abdominal obesity in coronary artery disease patients was specifically linked to reduced plasma adiponectin and elevated leptin levels [22], However, both studies focused on patient groups rather than healthy populations. Also, Gannage et al. observed that individuals with MetS had significantly lower adiponectin and higher leptin levels compared with those without MetS [13], Another study also introduced leptin and adiponectin as indicators of MetS [23], though both differed from the current study in that the participants were younger and MetS was diagnosed using stricter criteria for diagnosis. Rammuna et al. further documented that MetS was strongly associated with an increased leptin–adiponectin (LA) ratio, elevated leptin, and reduced adiponectin levels; however, their study applied more stringent diagnostic criteria for MetS and involved older participants [24]. Likewise, a 2020 study reported that higher adiponectin levels were linked to a reduced risk of MetS, whereas elevated leptin levels were associated with greater MetS risk [25], That study also included a larger population of older adults and employed stricter diagnostic criteria for MetS.

All lipid profile components, glycemic indices, blood pressure parameters, and waist circumference were significantly higher in the MetS group compared with healthy controls, except for serum HDL and insulin, which were higher in the control group. Similarly, an Eritrean study on elderly individuals reported associations between TC, non‐HDL cholesterol, and BMI with MetS [26]. Indian research also demonstrated a link with serum TG [27], whereas an Egyptian study confirmed significant associations between lipid profile components and MetS; it also observed a positive correlation between waist circumference and blood pressure [28]. Unlike previous studies that examined only individual metabolic parameters, this study evaluated MetS as a whole entity and demonstrated that the A/L remains a significant and reliable predictor even after stratification by BMI and age.

In the current study, a significant negative correlation was observed between serum adiponectin and TGs, as well as between the A/L and HbA1c. Additionally, there was a significant positive correlation between the A/L and HOMA‐B (Figure 3). Similar findings were reported [15], which indicated that the A/L ratio is more effective and relevant as a marker of insulin resistance compared with adiponectin or leptin alone. It was also found to be a more sensitive and reliable indicator than HOMA‐IR, especially as FPG levels increase in patients with T2DM. However, in the current study, no significant correlation was found between HOMA‐IR and adipokine levels.

Figure 4Correlations between serum galectin and adipokines with metabolic syndrome parameters in healthy (a) and metabolic syndrome (b) participants.

**Figure:**
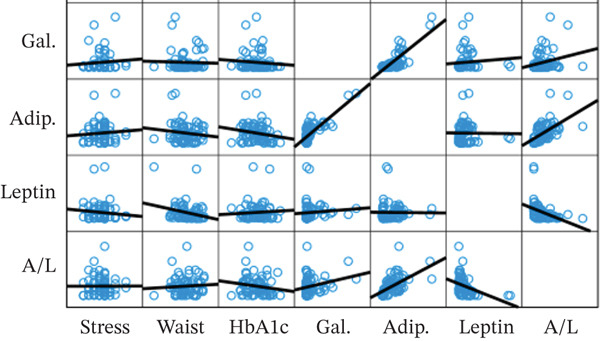
(a)

**Figure:**
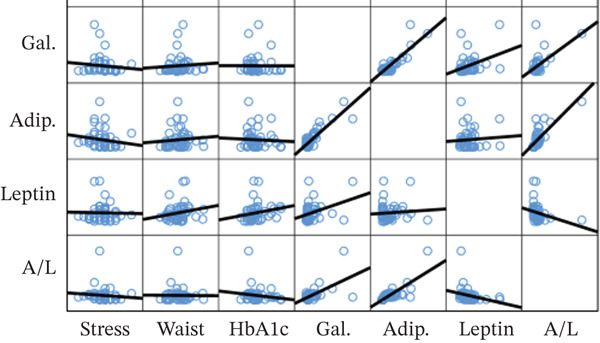
(b)

In this study, BMI was the only factor that significantly increased the likelihood of developing MetS in both young‐ and middle‐aged groups. Evidence from various populations also confirms this strong association. For example, research in Turkish adults has consistently shown that higher BMI is linked to an elevated risk of MetS [29]. In a large Chinese cohort, each 1 kg/m^2^ increase in BMI raised the odds of MetS by about 40% in both men and women [30], which is a comparable age group with the current study. Similarly, a meta‐analysis in Indian adults showed that obese individuals had nearly fivefold higher odds of MetS compared with those with normal BMI [31]. Together, these findings emphasize BMI as a powerful predictor of MetS across diverse populations.

The A/L emerged as the only significant predictor; with each one‐unit increase in this ratio, the odds of developing MetS decreased by 8.3%. Rammuana et al. reported that MetS cases with a mean age of 61 years were strongly linked to a higher L/A ratio, elevated leptin, and reduced adiponectin levels [24]. Several other studies also support these findings, highlighting the A/L as a valuable marker for diagnosing MetS. Other studies indicate that early metabolic disturbances in obese individuals are effectively reflected by the L/A ratio, underscoring its potential as a clinical biomarker for metabolic disorders [32], and the study by Chulaievska et al. (2010) examines the L/A ratio as a marker of cardiovascular risk in patients with MetS, with lower ratios indicating higher risk and metabolic complications [33]. Comparable outcomes were observed in children and adolescents, indicating that assessing the serum L/A ratio may be a valuable tool for evaluating obesity and its severity [34]. In postmenopausal women, this ratio presented as a marker of metabolic health, with elevated values frequently associated with a higher risk of metabolic issues, including insulin resistance [35], reinforcing the role of the A/L ratio as a key indicator of adipose tissue activity and a tool for identifying individuals at high risk of MetS.

This study has several limitations, including its cross‐sectional design that restricts the ability to establish causal relationships between adipokine levels, BMI, and MetS. Futhermore, a detail of lifestyle factors such as diet, physical activity, and stress, which are known to influence adipokine levels, were not comprehensively assessed. Other limitations were that it did not explore genetic, molecular, or longitudinal changes that might provide deeper insights into disease mechanisms. Future large‐scale, multicenter, longitudinal studies incorporating lifestyle and genetic variables are recommended to validate and extend these findings.

### 4.1. Clinical Implication

The clinical implications of the current study are that these biomarkers, serum galectin‐1 and adiponectin, along with the predictive value of the A/L ratio, could serve as accessible and noninvasive indicators for early identification of individuals at high risk of developing MetS, especially galectin‐1, which was merely studied in human serum.

Early detection using these markers may allow timely lifestyle interventions or pharmacological strategies to prevent progression to more severe metabolic or cardiovascular complications. Additionally, galectins, adiponectin, and leptin may represent potential therapeutic targets. Modulating these molecules, such as enhancing galectin‐1 or adiponectin activity or correcting leptin resistance, could improve insulin sensitivity, reduce chronic inflammation, and restore metabolic homeostasis [12, 32]. There are studies investigating the modulation of these adipokines to provide novel strategies for the prevention and management of MetS, complementing conventional approaches such as weight reduction, dietary modification, and increased physical activity [20, 24].

## 5. Conclusions

Serum galectin‐1 and adiponectin are significantly reduced in individuals with MetS, whereas the A/L emerges as the most reliable biomarker, outperforming individual adipokines and BMI in predicting metabolic risk. Taken together, the A/L represents a clinically relevant indicator for identifying individuals at high risk of MetS across different age and BMI groups.

## Author Contributions

Darya Saeed Abdulateef: conceptualization, data curation, formal analysis, methodology, resources, software, supervision, validation, visualization, writing—original draft, writing—review & editing. Trifa Abdalla Mahmood: conceptualization, data curation, investigation, methodology, project administration, resources, writing—review & editing. Lizan Ismail Arkawazi: conceptualization, data curation, investigation, resources, validation, writing—review & editing.

## Funding

No funding was received for this manuscript.

## Conflicts of Interest

The authors declare no conflicts of interest.

## Data Availability

The data that support the findings of this study are available on request from the corresponding author. The data are not publicly available due to privacy or ethical restrictions.
